# Applying Deep Neural Networks over Homomorphic Encrypted Medical Data

**DOI:** 10.1155/2020/3910250

**Published:** 2020-04-09

**Authors:** Anamaria Vizitiu, Cosmin Ioan Niƫă, Andrei Puiu, Constantin Suciu, Lucian Mihai Itu

**Affiliations:** ^1^Department of Automation and Information Technology, Transilvania University of Braşov, Braşov, Romania; ^2^Corporate Technology, Siemens SRL, Braşov, Romania

## Abstract

In recent years, powered by state-of-the-art achievements in a broad range of areas, machine learning has received considerable attention from the healthcare sector. Despite their ability to provide solutions within personalized medicine, strict regulations on the confidentiality of patient health information have in many cases hindered the adoption of deep learning-based solutions in clinical workflows. To allow for the processing of sensitive health information without disclosing the underlying data, we propose a solution based on fully homomorphic encryption (FHE). The considered encryption scheme, MORE (Matrix Operation for Randomization or Encryption), enables the computations within a neural network model to be directly performed on floating point data with a relatively small computational overhead. We consider the well-known MNIST digit recognition problem to evaluate the feasibility of the proposed method and show that performance does not decrease when deep learning is applied on MORE homomorphic data. To further evaluate the suitability of the method for healthcare applications, we first train a model on encrypted data to estimate the outputs of a whole-body circulation (WBC) hemodynamic model and then provide a solution for classifying encrypted X-ray coronary angiography medical images. The findings highlight the potential of the proposed privacy-preserving deep learning methods to outperform existing approaches by providing, within a reasonable amount of time, results equivalent to those achieved by unencrypted models. Lastly, we discuss the security implications of the encryption scheme and show that while the considered cryptosystem promotes efficiency and utility at a lower security level, it is still applicable in certain practical use cases.

## 1. Introduction

Over the recent years, machine learning algorithms, with emphasis on deep neural networks, have delivered remarkable solutions for personalized medicine, enabling customized diagnosis, treatment, and prevention [1]. Since deep neural networks are entirely data-driven systems that can learn explicitly from past experiences, they are commonly used as a way to integrate the knowledge and experience of medical experts into solutions for computer-aided detection (CADe).

To deliver results that are sufficiently reliable to be considered in clinical routines, machine learning-based solutions have to heavily rely on available medical data records [2]. However, as patient health information has some of the highest privacy requirements among all data types associated with an individual, its usability is greatly hindered. Moreover, given the fact that machine learning-based solutions require access to such sensitive information, concerns have recently been raised regarding data privacy and security [3]. Furthermore, the confidentiality protection laws currently adopted for the manipulation of personal data (e.g., EU GDPR and US HIPAA) demand the use of more effective privacy protection strategies.

Usually, a proper anonymization must be done in order to export sensitive data without violating confidentiality. Through data masking, some of the information properties are thus changed, resulting in a trade-off between confidentiality and utility. In certain cases, e.g., genomic data, the use of anonymized data limits the neural network's ability to gain valuable information and insight from the data. Herein, a method based on homomorphic encryption (HE) is employed as a way to address the limitations imposed by conventional methods, and to maintain confidentiality of biometric data. HE is a specific form of encryption which allows data to be encrypted while it is being manipulated. By preserving the mathematical structures that underline the data, HE represents a promising solution for guaranteeing privacy while still maintaining full utility. The chosen HE scheme (MORE) [4] allows for a limited set of operations to be conducted directly on encrypted data without exposing the underlying information or the encryption key. In the context of deep learning-based solutions, this property is especially useful as it ensures that both data and predictions are kept private while data are processed. Taking into account the practical difficulties arising from the use of deep networks on encrypted data and the inefficiency of current approaches, we propose a method that improves the effectiveness of encryption models in real-world applications by facilitating calculations over rational numbers, faster operations, and performance close to that achieved with an unencrypted model.

In order to evaluate the feasibility of the proposed privacy-preserving deep learning solution to produce reliable results, we consider a classic benchmarking application from the computer vision realm, i.e., digit recognition, and two personalized medicine applications. The herein proposed approach has been selected by the European Commission for the 2018 and 2019 Innovation Radar Prize Contest, whose goal is to identify Europe's top innovators and their innovations. In 2019, our solution has won the competition in the category “Industrial & Enabling Technologies” [5]. Parts of the work presented herein have been previously published in [6, 7]. They have been considerably extended herein to include a detailed description of privacy-preserving techniques for machine learning, homomorphic encryption, and deep learning. Furthermore, we have also included extensive details on the deep neural network models developed for the three applications that were considered, as well as detailed results in terms of model accuracy and runtime performance.

The remainder of the paper is structured as follows.  Section 2 discusses the latest achievements in the domain.  Section 3 includes a decryption of the considered homomorphic encryption scheme.  Section 4 offers a brief overview of deep learning and neural networks. Sections 5 and 6 address the proposed privacy-preserving pipeline in three deep learning applications.  Section 7 introduces the findings, with an emphasis on the correlation with the unencrypted counterpart. Finally, Section 8 draws the conclusions and outlines a set of issues that remain to be handled in future work.

## 2. Related Work

### 2.1. Privacy-Preserving Techniques for Machine Learning

In the past few years, great effort has been invested in the development of different privacy-preserving techniques with the potential of bridging the gap between data privacy and utility, demanded by the recent rise of privacy concerning scenarios. Among these methods, several privacy-preserving machine learning techniques, including homomorphic encryption (HE), secure multiparty computation (SMPC), and differential privacy (DP), have begun to advance rapidly. Such techniques ensure data privacy and at the same time allow for machine learning-based analysis to be performed. While these techniques have shown promising results, their adoption in modern machine learning applications remains bounded because they are highly dependent on the scenario. Moreover, there is always a trade-off involved between privacy and performance or between privacy and utility among these techniques, as each comes with specific strengths and vulnerabilities.

SMPC techniques provide a promising solution for data privacy by allowing analysis to be performed over sensitive data, distributed between different data providers, in a way that does not disclose the sensitive information beyond the analysis outcome. Although the idea of SMPC is not new, lately with the technological and hardware advances, more approaches using SMPC for data privacy guarantees in machine learning applications have emerged in the field [8–13]. The first attempt to train a neural network model in a SMPC setting has been made by Mohassel and Zhang [8], where the neural network-based analysis was performed inside a secure two-party computation for Boolean circuits via secret sharing, oblivious transfer, and garbled circuit. The greatest challenge in SMPC for machine learning is given by the computations of nonlinear functions as such operations introduce a high overhead in the training time. Moreover, the time needed for communications further limits their usability. Although great effort has been continuously invested in improving the technology, SMPC still implies a large communication overhead which makes it infeasible for machine learning, where a large amount of data is required. As the number of involved parties or the model complexity increases, the communication and computation costs are greatly affected.

An alternative solution is to use differential privacy which, as compared to SMP, implies a much lower computational complexity. Methods based on differential privacy provide good security and have been lately shown to achieve promising results when combined with machine learning techniques [14–22]. Such methods treat the privacy-preserving data analysis concerns by adding random noise into the algorithm at different stages. However, a proper noise injection mechanism is required to accomplish a reasonable trade-off between privacy and utility. Consequently, the added noise can greatly affect the precision of the machine learning-based analysis.

A few attempts have been made to address the challenge of data privacy-preserving in machine learning-based analysis through HE. This special type of encryption allows data to be encrypted while it is being manipulated. Hence, it aims at keeping the data private by allowing a third party to process the data in the encrypted form without having to reveal the underlying information. By preserving the mathematical structures that underline the data, HE represents a promising solution for guaranteeing privacy while still maintaining full utility. As ciphertext data are centralized to one single entity, it does not imply any communication bottleneck, as compared to SMPC. Although early work on HE [23] involves highly intensive computations, making the method infeasible for the machine learning algorithm, the recent subsequent schemes gave rise to a series of privacy-preserving machine learning solutions [24–29]. The first notable work that combines HE with neural network was proposed by Orlandi et al. [28]. The method uses a HE cryptosystem known to handle only a few basic operations directly on the ciphertext data. Therefore, to perform computations that cannot be completed in the encrypted domain, an interaction between the data owner and the server was required. Those operations were carried out using garbled circuit protocol (e.g., functions expressed as circuits of logical gates). CryptoNets [25] completely eliminate the interaction between the involved parties by using low-degree nonlinear polynomial functions. The method is based on the idea of using an already trained neural network on encrypted data to retrieve encrypted results. The encryption scheme used in CryptoNets, YASHE [30], does not support floating-point numbers. For this reason, real numbers were converted to integers. The computational complexity alongside the performance limitation introduced when handling large networks limits their usability. To mitigate the problem introduced by the model complexity, CryptoDL [26] proposed to approximate all nonlinear functions within a model with low-degree polynomials. However, none of these schemes cover privacy-preserving training in deep neural network models. The main drawback of these privacy-preserving neural network solutions is the computational overhead: deeper networks require more computations which results in longer running time. Additionally, the attempt to address the nonlinearity property in neural network models through an approximation mechanism does not necessarily result in better performances. In fact, most of these HE solutions fail to maintain the highest prediction accuracy due to the polynomially approximated activation functions. Moreover, due to the HE cryptosystems, these approaches involve an encoding mechanism, i.e., scaling, that converts floating-point numbers with fixed precision to integers.

Alternatively, many researchers began to combine these techniques to improve the level of accuracy and privacy [31–33]. Chase et al. [32] combined SMPC with DP techniques to enable privacy-preserving collaborative neural network training. The proposed solution provides data security guaranties through DP and uses SMPC to allow machine learning-based analysis when data are distributed between multiple parties. The experimental results showed that the performance was affected when larger networks were used. Barni et al. [31] provided a solution to enable computations within a neural network to be performed on homomorphically encrypted data but relied on the SMPC for the nonlinear functions. Gazelle [33] covers the privacy-preserving neural network inference phase by using HE for the linear operations and traditional SMPC (such as garbled circuits) for the activation function computation.

### 2.2. Homomorphic Encryption

With Gentry's first introduction of a fully homomorphic encryption (FHE) scheme [23], numerous variations of the original strategy were proposed in the literature [34]. Most of these schemes are known for their efficiency in terms of security, but they are computationally intensive and only a limited number of operations can be performed before decryption is no longer possible. This clearly restrains their usability in real-world applications. Aspects like computations being several orders of magnitude slower than the plaintext counterparts accumulated noise that limits the overall number of operations that can be performed, and all computations being implemented modulo *N* pose a great challenge for the synergy of deep learning and data analysis. While recent advances in HE led to many variants of encryption schemes, no currently available scheme can manipulate rational numbers.

Several open-source HE libraries have emerged in recent years, each one with different properties based on the employed encryption scheme [35]. Microsoft's Simple Encrypted Arithmetic Library (SEAL) [36] scheme, with support for the Brakerski/Fan-Vercauteren (BFV) [37] and the Cheon-Kim-Kim-Song (CKKS) scheme [38], and IBM's HELib [39] based on the Brakerski-Gentry-Vaikuntanathan (BGV) scheme [40] are two of the most widely used HE libraries. The first noticeable shortcoming of HELib is the lack of support for floating-point numbers. To allow for computations to be performed on rational numbers, SEAL takes advantage of a particular property of the CKKS scheme: rescaling can be performed without changing the encrypted value. Since a plaintext is represented as a polynomial with integer coefficients, floating-point parameters of the message are scaled by a parameter that affects the precision of the computations. Homomorphic operations performed with both HELib and SEAL introduce noise, thus limiting the number of operations that can be performed with ciphertexts. Noise-management techniques have been integrated to maintain the noise level below a certain threshold, such that the ciphertext does not become corrupted. While HELib uses the expensive procedure of bootstrapping to enable unlimited computations, SEAL uses a scale-invariant error reduction technique which requires an estimation of the number of operations that will be performed as an a priori information. Moreover, there are some limitations on the types of operations that can be performed on the ciphertext. The schemes used in HELib and SEAL are fully homomorphic with respect to addition and multiplication, and only polynomial functions can be easily performed. As a consequence, there is no implicit support for division, and nonlinear functions have to be approximated by low-degree polynomials.

While these schemes are known for their efficiency in terms of proven security, the above-mentioned restrictions, alongside their computational overhead, introduce noticeable constraints in the neural network topology, which in turn affect the performance of privacy-preserving neural networks [41].

Other proposed methodologies rely on employing partially homomorphic encryption (PHE) instead of FHE. Since FHE is currently practically impossible to be used in a real-world system, a viable approach is a system based on PHE that is specialized only for certain operations. Such an approach introduces a clear advantage in terms of running time and may be used in a practical application with reasonable overhead [42]. Various encryption schemes have homomorphic properties, out of which we mention the Paillier scheme [43], an additive homomorphic scheme where addition in the ciphertext space corresponds to multiplication in the plaintext space, and the ElGamal scheme [44], a multiplicative homomorphic scheme, which, through some modifications, can become additive. Other PHE with the potential to be used in a practical application are Goldwasser-Micaly [45] that allows computing the XOR operation on encrypted data, searchable encryption [46] with support for keyword search, order-preserving encryption [47] for sorting encrypted values, and deterministic encryption [43], that allows equality checks on encrypted values.

Another promising method is the algebra homomorphic encryption scheme (AHEE) [48], an encryption scheme that is homomorphic with respect to algebraic addition and multiplication, i.e., both addition and multiplications can be performed on encrypted data. A key advantage of this scheme is that it has a relatively small computational complexity, same as Paillier and ElGamal, while being homomorphic with both addition and multiplication. The main limitation of this scheme as well as for Paillier and ElGamal is that it only allows the encryption of relatively small integer numbers. More specifically, during the encryption process, an exponentiation operation needs to be evaluated, where the exponent is the message to be encrypted. Hence, even with a multiprecision arithmetic library, the operation can still cause an overflow. Using 1024 bit integers, we found that only numbers with up to about 10^3^ can be encrypted. This limitation becomes even more important when performing computations on encrypted data, i.e., one cannot determine if an encrypted number is too large for performing a certain operation.

In order to facilitate the privacy-preserving deep learning-based analysis, the cryptosystem must allow the computations within the model to be performed on rational numbers. To address this requirement, an encryption mechanism is typically used to encode a given rational number as a sequence of integers [49]. As some of the basic operations are difficult, if not impossible, to apply on encoded data, such an approach has limited functionality when applied on real data. Furthermore, the encoding strategy not only explicitly limits the data utility but also directly affects the results of the computations.

Over the past few years, many HE schemes have been proven to meet the security requirements. Although sufficiently secure, most of these approaches offer poor performance as they suffer greatly from runtime bloat, i.e., several orders of magnitude slower than the plaintext computations. This clearly restrains their usability in real-world applications. Consequently, simplified encryption schemes based on linear transformations emerged in the field as more practical alternatives. Despite the criticism for weaker security [50, 51], this type of cryptosystems seems to be the currently only feasible method with the potential to enable privacy-preserving computations in real-world applications.

As a consequence, the herein employed methodology relies on a variant of the matrix-based homomorphic encryption scheme proposed in 2012 by Kipnis and Hibshoosh [4]. In contrast with the currently adopted schemes in privacy-preserving neural network-based solutions [25, 26, 29], the MORE (Matrix Operation for Randomization or Encryption) encryption scheme is noise free and nondeterministic (multiple encryptions of the same plaintext data, with the same key, result in different ciphertexts). An unlimited number of operations can therefore be performed on ciphertext data. Moreover, the MORE scheme enables all four basic arithmetic operations over encrypted data. Herein, MORE was redesigned to directly support floating-point arithmetic in order to address the floating-point precision constraint of privacy-preserving deep learning-based analysis on real-world data.

## 3. Matrix-Based Data Randomization

A variant of the MORE encryption scheme has been considered and adapted to directly operate on floating-point data. Following the MORE encryption strategy, a plaintext scalar is encrypted as a *n* × *n* ciphertext matrix, and matrix algebra is employed to enable computations on ciphertext data. All operations performed on ciphertext data are therefore defined as matrix operations, e.g., the multiplication of plaintext scalars is formulated as the matrix multiplication of ciphertext matrices. The order of the matrix used to encrypt a message represents an important factor that governs the trade-off between security and efficiency. For a 2 × 2 setup, the MORE cryptosystem is summarized in Table 1.

The MORE scheme allows for algebraic operations to be performed on ciphertext matrices, i.e., given two encrypted matrices *C*_1_=*SM*_1_*S*^−1^ and *C*_2_=*SM*_2_*S*^−1^, for addition

(1) $$\begin{array}{c} C_{1}+C_{2}=SM_{1}S^{-1}+SM_{2}S^{-1}=S\left(M_{1}+M_{2}\right)S^{-1}, \end{array}$$

which is the encryption of *M*_1_+*M*_2_, and for multiplication

(2) $$\begin{array}{c} C_{1}C_{2}=SM_{1}S^{-1}SM_{2}S^{-1}=SM_{1}M_{2}S^{-1}. \end{array}$$

The same property applies to subtraction and division, as well as to plaintext scalar operations, making the scheme fully homomorphic for algebraic operations.

### 3.1. Encryption of Rational Numbers

The original MORE scheme, like any FHE or PHE approaches, is only applicable to positive integer numbers modulo *N*, with all operations being performed modulo *N*. To be able to operate on rational numbers, these schemes rely heavily on an encoding mechanism. As a consequence, a real number is converted into an integer or a set of integer numbers, and only afterwards the scheme is used to homomorphically encrypt the encoded number. A typical approach to formulate the encoding is through continued fractions [49]. Whilst a precise representation can be obtained, even basic operations on numbers expressed in this form are difficult to perform. Alternatively, a simpler encoding can be infused by multiplying the rational numbers with a large scaling factor. Although, more permissive, it requires a mechanism that manages the scaling factor, which is difficult to achieve for certain operations, e.g., division, where this factor is reduced. In addition, noise is typically introduced in the cryptosystem by extending the methodologies to operate on rational numbers. Consequently, a noise-management strategy has to be employed to limit the noise level. Even though the handling of rational numbers seems to be a straightforward problem, there is currently no solution that allows them to be used in the context of HE.

One of the main benefits of the MORE encryption scheme is that it can be directly formulated for rational numbers. The drawback is that the method becomes vulnerable to known ciphertext attacks, as described in Section 7.3.

### 3.2. Performing Operations over Encrypted Data

The MORE encryption scheme has been shown to be fully homomorphic with respect to basic algebraic operations. In real-world applications, including deep learning-based approaches, nonlinear (e.g., exponential, logarithmic, square root, etc.) functions need to be handled. Most of the regular approaches adopted to conduct nonlinear operations are based on the idea of approximating the specified function with a finite polynomial series (e.g., truncated Taylor series). Following this approach, nonlinear function computation relies solely on algebraic operations, being fully compliant with the MORE encryption setup. However, within the MORE cryptosystem, a more convenient approach is possible.

Given the properties that govern the encryption scheme, and knowing that ciphertext-based operations rely on matrix algebra, nonlinear functions can be computed either (i) directly as matrix functions or (ii) through matrix decomposition. While the first method is straightforward, the second approach is based on the property according to which a message *m*, that is to be encrypted, will be always found among the eigenvalues of the ciphertext matrix *C*. For example, in a 2 × 2 setup, one of the eigenvalues corresponds to the random number *r* used during the matrix construction, while the other corresponds to the message *m*. To ensure that the message can only be identified through proper decryption and by possessing the secret key, the random number *r* is generally chosen to be statistically indiscernible from the message. Applying a function *f* on the ciphertext data *C* is therefore equivalent to applying *f* directly on the eigenvalues of *C*. Thus, matrix decomposition *VLV*^−1^ is first used to decompose the ciphertext matrix *C* into eigenvalues *L* and eigenvectors *V*. Thereafter, the nonlinear function that is to be evaluated is applied independently on each of the eigenvalues. Finally, the resultant ciphertext matrix is reconstructed as *C*_*f*_=*Vf*(*L*)*V*^−1^ using the original eigenvectors and the eigenvalues evaluated on the function *f*. As compared to the direct matrix function-based computations, this approach can even be used to perform comparisons between the ciphertext matrix *C* and a plain scalar *s*. Neither of these two methods support nonlinear binary operations between two ciphertext data. However, in deep learning, such operations can be completely avoided.

Starting from these strategies, Algorithm 1 shows how, given any ciphertext *C* ∈ *ℝ*^2×2^, the two proposed methods can be used to formulate the function *f*(*x*)=(1/(1+*e*^−*x*^)) defined on *x* ∈ *ℝ*, under the MORE assumptions. This function is known as the logistic sigmoid function and is widely used in neural networks for its nonlinear properties, as will be outlined in the next sections.

## 4. Deep Learning

Since their first appearance in 1943 [52], the functionality of neural networks has been constantly associated with the way people learn and process information. More specifically, they were designed to emulate the synaptic connections between brain neurons and later on became the foundation of deep learning.

Fueled by the recent hardware advances, the rise of big data and desire for exceeding human-level performances, deep neural networks are currently becoming a widespread technology in data mining. The most notable turn point was marked in 2012 [53], when a deep learning-based approach, AlexNet, obtained unprecedented results (11.5% error rate) in computer vision on the ImageNet Large-Scale Visual Recognition Challenge [54], surpassing the winning entry of 2011 by an improvement of ≈45%. Thereafter, the ImageNet classification accuracy gradually improved; in 2017, the lowest reported error rate on ImageNet has dropped to 2.25% [55], being superior to the reported human error rate of approximately 5% [54]. Since then, deep neural networks experienced an extraordinary growth rate, leading to an explosion of research in many fields.

Medical data interpretation is highly subjective, prone to errors, and most often it depends on the experience of the medical expert. To automatically capture the high variability in anatomical structures and features, deeper neural networks are required. As compared to traditional machine learning models, deep networks can model the complex relationships and capture relevant information and patterns from different perspectives, representing a much better candidate for the development of CADe solutions [1]. In consequence, nowadays, deep learning is being widely used for tasks that were previously known to represent great challenges, achieving state-of-the-art performances in different healthcare domains and applications [1].

### 4.1. Neural Networks

**Figure 1:**
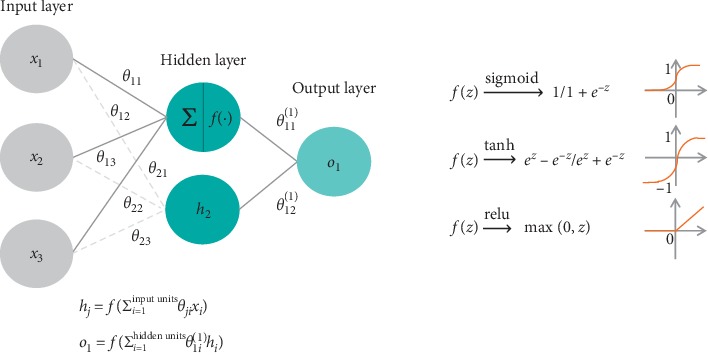
Architecture of a simple neural network described by the input, output, and a hidden layer in between. Information flows through all layers, starting from the input layer to the output layer. Herein, every neuron receives information from all neurons of the previous layer (in literature called a fully connected layer). The connections *θ* between the processing blocks are the parameters that have to be adjusted in accordance with data and the formulated problem. Each processing block performs a transformation (herein a weighted sum of the input parameters) and the result is passed to an activation function *f* that will be used to add nonlinear properties in the network. Activation functions are usually selected from a set of limited functions with certain mathematical properties. Nonlinearity is needed to allow for a complex arbitrary functional mapping between input and the output data.

On a high level, a neural network can be defined as a computational model that maps inputs to outputs through a composition of layers with interconnected processing blocks (transformations and activation functions). The architecture of a simple neural network is depicted in Figure 1. To allow for a complex arbitrary functional mapping, nonlinear activation functions are typically added at each processing block. They filter the information that passes through the network, determining what input signal is relevant to be forwarded to the following layer. Essentially, they decide whether a certain neuron should be activated or not and without them the neural network becomes a simple linear model. There are several functions used as activation functions, including but not limited to logistic sigmoid, hyperbolic tangent (tanh), and rectified linear unit (ReLU).

In supervised learning, the model automatically learns the mapping function (parameters of the model) based on labeled training examples in an iterative fashion by gradually making an adjustment with the effect of minimizing an error function, i.e., loss, between expected and achieved outputs.  Algorithm 2 describes the typical operations involved in the neural network training phase. The term epoch refers to the complete processing of the entire training dataset. Due to computational reasoning, for large datasets, the processing, and hence the parameter adjustment, is done on subsets of data (batches). In the first iteration, the forward propagation step provides the predicted outputs for the input samples given a set of randomly initialized parameters. Thereafter, the error (loss) between the predicted outputs and the desired ones is computed and passed backwards through the network to determine the direction in which each parameter has to be adjusted to decrease the overall prediction error. Finally, the obtained gradients are used to update the network parameters following a numerical optimization method (e.g., gradient descent). This process is repeated over many epochs until the network error stops decreasing.

Upon training, the network should be able to provide results which are statistically similar to the expected ones even when presented with input data never encountered by the network during training. Consequently, neural networks can be used in predicting an output from certain input features, classifying data and even localizing patterns or objects in images.

### 4.2. Going Deeper: Deep Neural Networks

In essence, a deep neural network is nothing else than a neural network model composed of several layers of processing blocks and organized as an input layer, followed by multiple hidden layers, and an output layer. Over the years, it has been shown that such an architecture facilitates the modeling of highly complex functions, allowing for the learning of richer intermediate representations. Hence, the key difference between shallow and deep neural networks is given by the depth of the models; although not standardized, typically a network with depth higher than two falls into the deep learning category.

Over the past few years, we have seen a global trend for neural networks becoming deeper. Within 3 years, reported state-of-the-art models experienced a massive increase in depth, from the 8 layers of the AlexNet [53] to 100+ layers found in residual networks [56]. This contradicts the universal approximation theorem according to which a neural network with a single wide hidden layer is already enough to approximate any function [57]. While in theory, the same mapping function between input and output may be learned by shallow and deep networks, empirical work showed that it is harder to optimize a shallow network to approximate a complex function as accurate as deep networks, even when the same number of parameters is used in both models [58]. By using deep architectures, the learned function is expressed as a composition of several simple functions. It was previously shown [59] that the same degree of accuracy can be achieved by shallow and deep networks in approximating a function using the property of compositionality, but exponentially lower number of training parameters and sample complexity were required by the deeper models. When AlexNet won the ImageNet competition, it has been reported that removing any of the hidden layers led to a 2% loss in performance [53]. Moreover, as emphasized by Goodfellow at al. [57], a deeper model can reduce the generalization error. This statement is backed by the results obtained in practice in many fields and tasks where a greater depth was associated with better generalization [60–63]. These results suggest that deeper networks are more powerful at encoding the multilevel feature representation directly from the high dimensional input data.

However, the training of deep networks is challenging. As the network becomes deeper, problems such as vanishing or exploding gradients may appear, which hamper convergence and degrade the model performance [64]. These challenges have been largely addressed by the deep learning community leading to improvements in the architectures by introducing intermediate normalization layers [65] and shortcut connections [56]. Moreover, deeper networks require substantially more computing power, which sometimes leads to a trade-off between model complexity and performance. As in any data-driven model, the training dataset represents the fundamental resource behind the achieved performance, and deeper networks require a considerably larger amount of training examples to generalize well. While the performance of traditional machine learning approaches saturates once a certain training dataset size is reached, deep learning models have the capability to further improve their performance with more data.

### 4.3. Deep Learning Models

The deep learning model that won the ImageNet competition in 2012 was not a traditional deep neural network. Instead, a deep convolutional neural network (CNN) architecture was proposed to enable feature learning directly from the input images, completely mitigating the need for hand-designed features as in traditional learning-based models. In a CNN, the meaningful contents for a specific task, usually described as high-level features, are learned from the lower ones in a fully automatic manner incorporated in the backpropagation-based training procedure. Since its inception, CNN remains one of the most popular types of deep learning approaches used in data-driven medical imaging analysis [66].

Compared to a traditional deep neural network, where all layers are fully connected, a CNN relies extensively on convolution and pooling layers (downsampling units). The operation performed inside a convolutional layer is a dot product between the input of the layer and a small learnable filter (kernel). To cover the entire input, a sliding window strategy is adopted. During training, the parameters of the filters are adjusted to extract relevant information for the given task directly from the input data. Pooling layers have no learnable parameters and are used to reduce the spatial dimension of the data by reducing the information in a small area to a single value (by performing an averaging or maximum operation). By using a combination of such layers, the network exploits local connectivity making the model invariant to scaling or shifting transformations. By increasing the number of layers, the network's receptive field is expanded, which in turn forces the model to progressively capture more complex patterns, from edges to shapes or objects. Moreover, the use of local receptive fields, sparse connectivity, and parameter sharing drastically reduces computational overhead and the number of parameters that have to be learned, as compared to traditional neural networks.

Over the past few years, based on the connection pattern between layers, many neural network architectures have been proposed for specific tasks: fully connected neural networks (FCNNs) are primarily used for classification or regression tasks where a global relationship between the input features is required and spatial information can be discarded, CNN specialized on data that pose a certain spatial relationship (e.g., images), and recurrent neural networks (RNNs) are used for sequence or time-series data processing.

**Figure 2:**
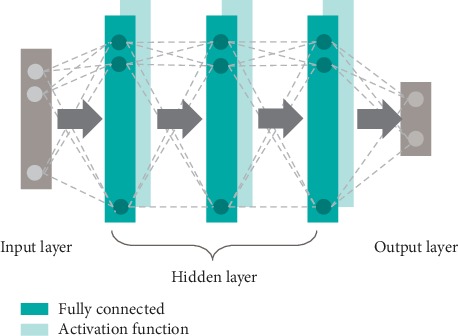
General structure of a deep fully connected or feed-forward neural network (FCN). The data are fed through a stack of fully connected layers, where each neuron is connected to all neurons of the following layer. The network is typically used on tabular data or on handcrafted features.

**Figure 3:**
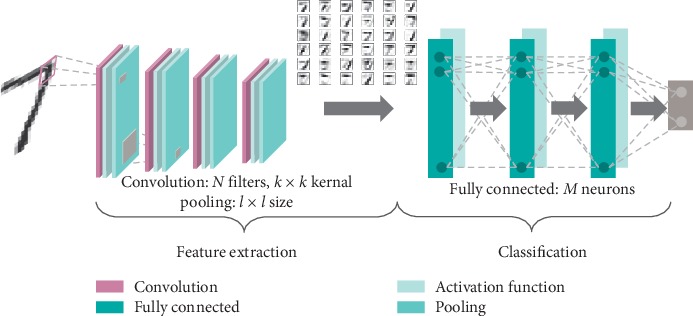
General structure of a deep convolutional neural network (CNN). The network involves two main parts: a feature extractor and a classifier. The feature extractor is composed of multiple alternating convolutions and pooling layers. Each convolution layer performs a full scan of its input by gradually analyzing small regions (*k* × *k*) and extracting a number *N* of distinctive features. The extracted information is further passed to the pooling layer where its dimensionality is reduced based on the pooling size (*l* × *l*). With each additional layer, the network learns to extract higher-level features while the dimensionality of each feature is continuously reduced. The classifier takes the final extracted features as input to a fully connected neural network that combines the information to produce the final output. Such a network is primarily used for processing images or videos.

Motivated by the input data types, the amount of available data, and the formulated problems, herein we focus on a FCNN for solving a regression task and on a CNN for image-based analysis. The general architectures of the deep neural networks underlying the studied methods are represented in Figures 2 and 3.

## 5. Deep Neural Networks over Encrypted Data

In this section, aspects of privacy-preserving deep neural networks are described. The proposed method relies on the MORE homomorphic encryption scheme and enables both the training and the exploitation of classical neural network models directly on homomorphically encrypted data.

### 5.1. Method

Over the past few years, we have seen remarkable performances being obtained by deep learning-based analysis in the medical field. The complex mathematical formulation of deep learning models ultimately breaks down to a series of repeating blocks of computations that rely on a limited set of simple operations over rational numbers. In fact, many of the deep learning-based state-of-the-art results have been achieved by deep neural network models that were using only limited types of operations (e.g., multiplication, addition, division, subtraction, exponential and logarithm). By leveraging the homomorphic property of the MORE scheme, the functionality of neural network models can be further formulated to account for operations on ciphertext data.

**Figure 4:**
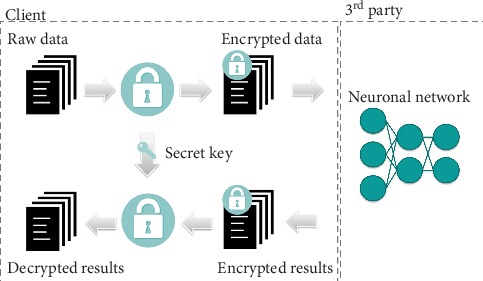
Workflow of the proposed privacy-preserving deep learning-based application relying on homomorphic encryption.

The proposed workflow, based on HE and deep learning, is outlined in Figure 4. Before being processed, training data are encrypted with a secret key that is never shared (Algorithm 3). Thereafter, the deep learning-based model will have access only to the encrypted version of the data (ciphertext), while the actual data (plaintext) are detached from the processing unit and remain private on the side of the data provider. Finally, with the homomorphic property underlying the MORE encryption scheme, the direct support for floating-point arithmetic, and with all operations performed inside the network formulated to ensure applicability on ciphertext data, the network can be trained directly on ciphertext data following the classical pipeline described in Algorithm 2. This results in a model that provides encrypted predictions, which can only be decrypted by the owner of the secret key, following Algorithm 4. Once the training phase is finalized, the encrypted form of a model can be employed to predict new encrypted instances (inference phase), where input samples are encrypted with the same key as the one used during the training phase. The MORE cryptosystem relies on symmetric keys. Hence, a secret key, generated following the approach presented in Algorithm 5, is used for both the encryption of the plaintext data and the decryption of the ciphertext data.

The proposed deep learning-based ciphertext data analysis framework is presented in Algorithm 6. For the sake of comparison and validation, we also provide the pipeline used for plaintext data analysis (Algorithm 7). Note that in Algorithm 6, all operations performed during training and prediction are formulated in accordance with Sections 3.1 and 3.2.

Following this approach, data privacy is retained during both training and inference, as the external party operates explicitly on ciphertext data and delivers results as ciphertext data. Consequently, the secure processing of medical data is performed in such a way that the external party cannot derive knowledge from the patient data, and the user is unable to obtain information regarding the machine learning model [7].

## 6. Experiments

To validate the proposed method, we focused on solving three types of deep learning applications: regression, binary, and multiclass classification. We first addressed a well-known benchmarking application (digit classification) and then aimed at addressing the privacy issue in two healthcare related applications by training neural network models on encrypted data (i) to assess whole-body hemodynamics and (ii) to distinguish coronary artery angiographic views.

The aim of the conducted experiments was not to achieve deep learning-based state-of-the art results for the proposed problems but to investigate the possibility of maintaining data privacy while still allowing for computations within a neural network to be successfully performed over the encrypted version of the data.

This section describes the datasets and the experimental setup, including the proposed deep neural network models and the common hyperparameters used for each of the above formulated problems.

### 6.1. Problem Formulation

#### 6.1.1. MNIST: A Typical Dataset for Neural Networks

A typical problem studied in the context of neural networks is that of classification. More specifically, the problem of image categorization is in accordance to the information depicted in the image. The MNIST (Modified National Institute of Standards and Technology) database [67] contains images representing handwritten digits and is typically employed as reference for benchmarking image classification algorithms.

The choice for the digit recognition task as a first experiment that addresses the challenges of privacy-preserving computations in neural network models was made with the intention of providing valuable insights about the strengths and vulnerabilities of the proposed method in a task that is already considered solved.

The approaches used in the literature to solve the digit recognition problem range from the classical linear classifier, to support vector machine (SVM), and more recently to deep neural network models. However, deep CNN models have been shown to perform significantly better than other types of classifiers on MNIST, leading to the lowest reported test error rates [68]. Moreover, when corresponding shallow networks were employed, the error rate has increased [68], reemphasizing the need for deeper models.

The digit recognition problem is framed as predicting the probability of an image belonging to each of the 10 classes (0–9 digits). Hence, target labels are typically represented as one-hot vectors with only the associated class having the value 1 and 0 for the rest. This example is a typical case of a multiclass classification problem (*C*=10 classes) that can be solved by a neural network model trained to minimize a cross entropy error between the predicted $\left(\tilde{y}\right)$ and expected (*y*) probability distributions:

(3) $$\begin{array}{c} CE\left(y,\tilde{y}\right)=-\sum_{c=1}^{C=10}y_{i}\log\left({\tilde{y}}_{i}\right). \end{array}$$

**Figure 5:**
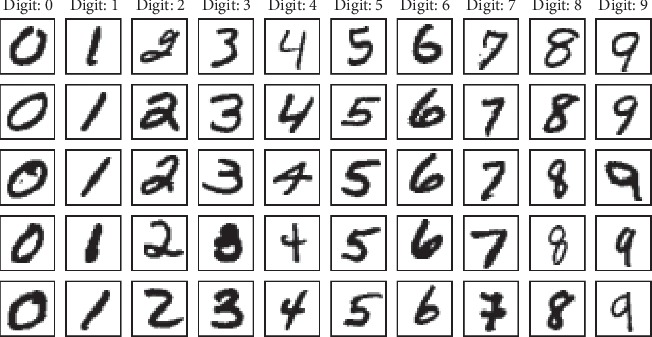
Example images from MNIST dataset.

*(1) Dataset*. The MNIST dataset consists of 70,000 gray-scale images of relatively small dimension, 28 × 28, each image being labeled with the digit it depicts (Figure 5). The digits are size-normalized and centered in the images. The MNIST samples were partitioned into three datasets resulting in 50,000 cases used for training a neural network classifier, 10,000 for validating the trained model, and 10,000 for assessing the classifier's performance. The training samples were balanced over the ten classes to avoid class imbalance problems that commonly arise in classification.

In MNIST images, pixel values range from 0 to 255. In order to improve training convergence, pixel values were scaled to [0,1] based on the minimum and maximum pixel intensity. To perform the neural network training, the MNIST labels represented by numerical values from 0 to 9 were encoded into categorical values as one-hot vectors. Therefore, each digit was represented by a vector of length equal to the number of classes, and where the digit position was marked in the vector with a value of 1, all other values being set to 0.

#### 6.1.2. Whole-Body Circulation Model

**Figure 6:**
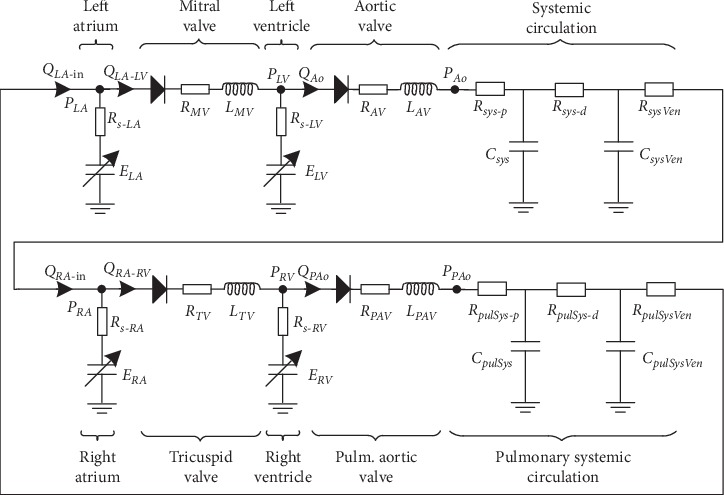
Whole-body circulation model.

In the following we showcase the use of the considered approach in a personalized medicine application, based on a whole-body circulation model (WBC). The cardiovascular system is a closed loop system and cannot be modeled using spatial hemodynamic models (3D models especially) due to the associated large computational complexity. Thus, the typical approach is to employ lumped parameter models, which rely on the analogy between electricity and hydraulics. The model considered for this study is depicted in Figure 6 and is composed of the left and right ventricles and atria, the arteries, capillaries, and veins of the systemic circulation, and the arteries, capillaries, and veins of the pulmonary circulation [69].

Each of the four heart chambers is represented by a time-varying elastance model:

(4) $$\begin{array}{c} P\left(t\right)=E\left(t\right)\cdot \left(V\left(t\right)-V_{0}\right)-R_{S}Q\left(t\right), \end{array}$$

where the time-varying elastance is *E*, the chamber volume is *V*, the dead volume of the chamber is *V*_0_, and an additional term accounts for the relationship between cavity pressure and flow, parameterized by *R*_*s*_ [70] (*R*_*s*_=*K*_*s*_*E*(*t*)(*V*(*t*) − *V*_0_),  *K*_*s*_ − constant). The chamber volume is computed as

(5) $$\begin{array}{c} \frac{\text{d}V}{\text{d}t}=Q_{\text{in}}-Q_{\text{out}}. \end{array}$$

The four heart valves (aortic, mitral, pulmonary, and tricuspid) are modeled using a diode, a resistance, and an inertance. The valve closure and opening is triggered by the upstream-downstream pressure difference. The following hemodynamic relationship is employed when the valve is open (flow is zero when the valve is closed):

(6) $$\begin{array}{c} P_{\text{in}}-P_{\text{out}}=R_{\text{valve}}\cdot Q+L_{\text{valve}}\cdot \frac{\text{d}V}{\text{d}t}, \end{array}$$

where *P*_in_ is the pressure at the inlet of the valve and *P*_out_ is the pressure at the outlet of the valve. The valve is opened when *P*_in_ becomes larger than *P*_out_ and is closed when a negative flow rate is encountered. The systemic circulation is represented by a three-element Windkessel model:

(7) $$\begin{array}{c} \frac{\text{d}P_{Ao}}{\text{d}t}=R_{sys-p}\frac{\text{d}Q_{Ao}}{\text{d}t}-\frac{P_{Ao}-P_{ven}}{R_{sys-d}\cdot C_{sys}}+\frac{Q_{Ao}\left(R_{sys-p}-R_{sys-d}\right)}{R_{sys-d}\cdot C_{sys}}, \end{array}$$

where the distal and proximal resistances are *R*_*sys*−*p*_ and *R*_*sys*−*d*_, respectively, the compliance is *C*_*sys*_, and the venous pressure is *P*_*ven*_. The systemic venous circulation is represented by a two-element Windkessel model:

(8) $$\begin{array}{c} \frac{\text{d}P_{ven}}{\text{d}t}=\frac{Q_{ven}}{C_{sysVen}}-\frac{dP_{ven}-P_{RA}}{R_{sysVen}\cdot C_{sysVen}}. \end{array}$$

The pulmonary circulation is modeled analogously.

When run under personalized conditions, this hemodynamic model can determine different quantities which are clinically relevant, namely, the arterial compliance, the arterial resistance, the dead volume of the heart chambers, the stroke work, the arterial/ventricular/atrial elastance, the ventricular-arterial coupling, the PV loop, etc. To personalize the model, its parameters need to be calibrated, to ensure that patient-specific measurements are matched by the model outputs.

The personalization procedure considered herein has been previously introduced in [71]. It relies on two steps: (i) a number of parameters are computed from the input measurements and (ii) an automated calibration method, relying on an optimization based iterative workflow, tunes the remaining parameter values.

The input measurements are as follows:

- Pulmonary circulation: systolic pressure in the pulmonary artery, end-diastolic pressure in the pulmonary artery, end-diastolic and end-systolic volumes of the right ventricle, and ejection time of the right ventricle.
- Systemic circulation: systolic pressure in the aorta, end-diastolic pressure in the aorta, end-diastolic and end-systolic volumes of the left ventricle, and ejection time of the left ventricle.

The patient-specific outputs computed after finalizing the personalization are as follows:

- Pulmonary circulation: right ventricular dead volume, resistance and compliance of the pulmonary arterial circulation, and ratio between proximal and distal resistance in the pulmonary arterial circulation.
- Systemic circulation: left ventricular dead volume, resistance and compliance of the systemic arterial circulation, and ratio between proximal and distal resistance in the systemic arterial circulation.

The automated personalization procedure described above is formalized as an optimization problem, having as goal the determination of a set of parameter values which minimize the difference between computed and reference objective values. The number of objectives is chosen to be equal to the number of parameters to be personalized; hence, we are solving a system of nonlinear equations. Concretely, the dogleg trust region method is employed for finding the root of the system of equations [71].

Although one WBC forward run is very efficient in terms of computation time, the hundreds of runs required for the calibration procedure lead to a runtime of up to one minute on a standard desktop hardware configuration. Hence, a solution capable of determining in real time the personalized hemodynamic measures of interest of the WBC model would be very valuable, even when considering only the plaintext version.

In the context of deep neural network, this problem is framed as predicting real-valued quantities from a set of input parameters. By modeling this regression task using neural network models, the parameters are adjusted during training to minimize the squared differences between the predicted ($\tilde{y}$) and expected target (*y*) values:

(9) $$\begin{array}{c} \text{MSE}\left(y,\tilde{y}\right)=\frac{1}{2n}\sum_{i=1}^{n}{\left(y_{i}-{\tilde{y}}_{i}\right)}^{2}, \end{array}$$

where *n* is the number of real-valued quantities.

**Figure 7:**
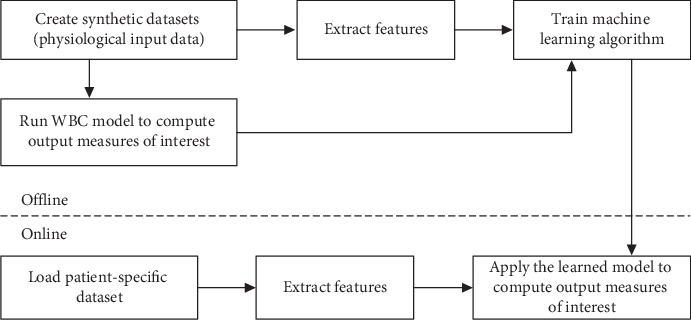
Proposed workflow of the deep learning-based model.

*(1) Dataset*. A large training database is required for the deep learning based approach. Since such a database was not available, we have resorted to an approach that we have successfully employed in the past for diagnosing in real time coronary stenoses from computer tomography data [72]: synthetic data are generated, and the training of the deep neural network is based only on synthetic data. The calibration framework is run for each synthetic dataset and the hemodynamic model is employed to compute the measures of interest. In the prediction phase, actual patient data are used as input and the patient-specific measures of interest are determined by employing the learned model (Figure 7).

We generated a training dataset containing 10000 synthetic samples, covering a wide range of functional and anatomical variations that can be identified in patients and in a healthy population [73]. For each sample, the input data were represented by 9 parameters, and the WBC model computed 12 measures of interest as described above. The 10000 synthetic samples were divided as follows: 7000 for training, 1000 for validation, and 2000 were used for the final test.

#### 6.1.3. X-Ray Coronary Angiographies

The main imaging modality for the diagnosis of coronary artery disease (CAD) is invasive X-ray coronary angiography (ICA) [74]. It allows for a comprehensive assessment of both the function and the structure of the heart. During the invasive procedure, a dye with radio opaque characteristics is inserted in the coronary vessels and a set of images is recorded in succession by an X-ray scanner. This offers a comprehensive overview of the coronary trees, allowing for an evaluation of the coronary stenosis severity, which may be performed either quantitatively (QCA—quantitative coronary angiography) or qualitatively (visual assessment) [75].

**Figure 8:**
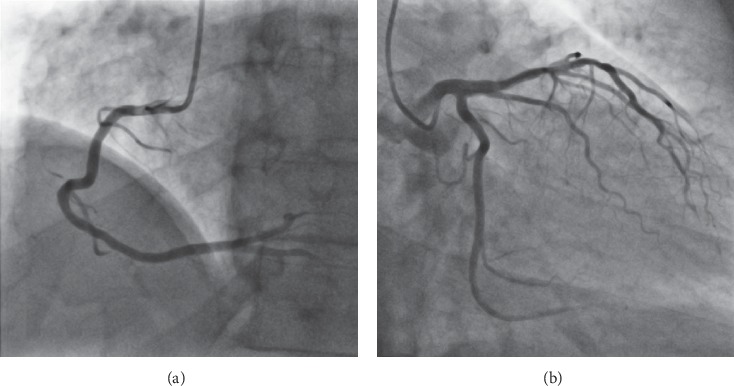
(a) Right coronary artery. (b) Left coronary artery.

However, a solely anatomical evaluation of the stenoses has limited accuracy, and hence a diagnostic index performing a functional assessment (FFR—fractional flow reserve) has been proposed as a superior approach in terms of long-term outcome [76]. Recently, methods for computing FFR directly from the angiographic images have been also introduced [72, 77, 78]. In all cases, angiographic images are acquired independently for the left and the right coronary artery (LCA and RCA) (Figure 8).

Thus, an active research topic in the coronary artery assessment is the automated processing of angiographic images [79], having as goals

- Automated assessment of the coronary stenosis degree.
- Image-based computation of functional diagnostic indices [72, 78].
- Automated composition of medical reports starting from the findings in the angiographic images.

These represent just a subset of the use cases based on coronary angiography, where an accurate LCA/RCA view classification is a crucial prerequisite for the subsequent processing steps.

**Figure 9:**
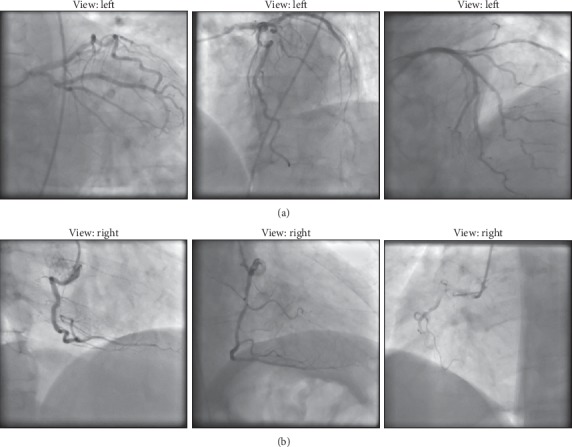
X-ray coronary angiography training samples.

The X-ray coronary angiography view recognition can be formulated as a binary classification problem, where a neural network model learns to predict the probability of an image belonging to the positive class (represented by the value 1). Hence, the two categories, LCA and RCA, can be encoded as 0 and 1, respectively. This translates to training a deep neural network in a supervised manner, to minimize a binary cross entropy error:

(10) $$\begin{array}{c} \text{BCE}\left(y,\tilde{y}\right)=-\sum_{c=1}^{C=2}y_{i}\log\left({\tilde{y}}_{i}\right)=-\left(y\;\log\left(\tilde{y}\right)+\left(1-y\right)\log\left(1-\tilde{y}\right)\right., \end{array}$$

where *y* represents the expected class label and $\tilde{y}$ is the predicted probability (as output by the classifier) of the input image as being an RCA image. Hence, the output value of the classifier must reside between 0 and 1 (Figure 9).

*(1) Dataset*. To investigate the feasibility and effectiveness of deep learning-based analysis for X-ray coronary angiography view classification, an in-house database was used. The available database consisted of 3378 coronary angiographies with 512 × 512 pixel resolution. A manual annotation of the images was performed to determine the ground truth LCA/RCA view information. One frame, well contrasted by the injected dye, was considered for each angiographic image [9].

To conduct learning-based experiments, the database was split at patient level into training, validation, and test sets. Thus, the training set consisted of 1996 images, the validation set of 680 images, and the testing set, employed only for the end evaluation of the model, consisted of 702 images. Balancing was performed in all 3 sets (prevalence of 1 : 1 for the RCA and LCA images).

Due to privacy considerations, there is a well-known lack of publicly available databases in the medical field. Therefore, to increase the amount of training samples, we opted to synthetically infuse new images through data augmentation. Offline augmentation was employed, leading to a four-fold increase of the training dataset size. The considered strategies relied on transformations like rotation (±10 degrees), zooming, and shifting. Moreover, the coronary angiography images were downsampled by a factor of 2, resulting in a 256 × 256 pixel resolution. The range of the input pixel values was rescaled to [0,1] based on the min-max normalization strategy. Hence, given a pixel value *x* and the minimum and maximum pixel values across the image (min_*x*_, max_*x*_), the normalized value was computed as ((*x* − min_*x*_)/(max_*x*_ − min_*x*_)). Based on annotations, the output, i.e., target, was assigned with one of the two values: 1 for RCA and 0 for LCA.

### 6.2. Ciphertext Database Preparation

To evaluate the performance of the proposed privacy-preserving method, three databases have been used: images of handwritten digits (MNIST), X-ray coronary angiographies, and synthetically generated WBC samples. A brief overview of these databases is given in Table 2. More details can be found in Sections 6.1.1, 6.1.2, and 6.1.3.

To address the challenge of privacy-preserving computations and to evaluate the use of deep neural network models over encrypted data, for each dataset, the input samples, i.e., image or feature vectors, were encrypted following the MORE encryption strategy, as described in Algorithm 3. Similarly, the target values, i.e., class labels or real-valued quantities, were also encrypted, except for the binary classification problem where the target was given as plaintext. We chose to encrypt only the input data, i.e., the coronary angiography images, and leave the target, i.e., binary label 0 or 1, as plaintext to show that training can as well be performed if labels are formulated as plaintext. For each experiment, a different secret key was generated following Algorithm 5. In the preprocessing step, the input and output features were normalized, i.e., using mean and standard deviation or minimum and maximum values, to achieve faster convergence.

### 6.3. Deep Neural Network Model Architecture

To assess the feasibility and effectiveness of deep neural networks to operate directly on homomorphically encrypted data, we conducted three experiments and trained (i) a CNN for digit recognition on encrypted handwritten images, (ii) a traditional FCNN for real-time hemodynamic analysis, where both the input feature vector and the ground truth outputs were encrypted, and (iii) a CNN for encrypted X-ray coronary angiography view classification. For a comparison of model performance and convergence, we also trained the counterpart models on plaintext data.

Although more efficient alternative deep neural network models (e.g., improved activation functions and greater depth) can be adopted to ensure better convergence and superior performance, herein the purpose of the experiments was to assess the correctness and effectiveness of different deep neural network models operating on ciphertext data, as compared to the counterpart models trained on plaintext data.

#### 6.3.1. Deep Neural Network for Handwritten Digit Classification

Starting from the latest results obtained by CNN models on the MNIST digit recognition task, a CNN was employed on encrypted input-output value pairs. The topology of the proposed privacy-preserving CNN is described in Table 3.

The input image represented as ciphertext data is passed through a stack of 2 convolution layers where 8 and 16 filters with small 3 × 3 receptive fields were used to extract hierarchical image features. Through averaging, the pooling layer downsamples the images by a factor of two. The last two fully connected layers cover 100 and 10 nodes, respectively, and all activation functions employed in the network, except for the ones in the last layer, are sigmoid functions. Although more efficient options exist to introduce nonlinearities in the network, herein we focused on sigmoid as it is one of the most frequently used activation function. To convert the two-dimensional matrix of features into a vector, a flat layer has been added between the last convolutional layer and the first fully connected layer. A softmax activation function was considered in the output layer to provide class probabilities. Given an input value *y*_*i*_, the softmax function converts the value into a probability and ensures that output probabilities sum to 1: *S*(*y*_*i*_)=(*e*^*y*_*i*_^/∑_*j*=1_^10^*e*^*y*_*j*_^).

#### 6.3.2. Deep Neural Network for Real-Time Hemodynamic Analysis

Given the nature of the input data, i.e., information represented as a feature vector, and driven by the need to model the decision of the network based on a global dependency between input features, a fully connected neural network with 3 hidden layers was employed. The topology of the proposed privacy-preserving FCNN is described in Table 4.

Although deeper and wider networks could have been considered, in the absence of a larger training dataset, this would have led to overfitting. Hence, the number of layers and units was chosen to facilitate the generalization capability of the network. Both the logistic sigmoid and the hyperbolic tangent (tanh) functions were chosen as nonlinearities, with all 3 hidden layers holding the same number of neurons [40]. Herein, a hyperbolic tangent activation function was used to facilitate training and decrease the risk of vanishing gradients. Moreover, we show that neural networks over ciphertext data are not bounded to only one type of nonlinear functions, i.e., sigmoid, and that more functions can be adopted if the involved operations are supported by the MORE encryption scheme. Since the problem being solved is formulated as a regression task, no activation function was specified in the output layer, every output value being a linear combination of the outgoing values of the last hidden layer. This ensures that the outputs are real values ranging from [−*∞*, +*∞*].

#### 6.3.3. Deep Neural Network for View Classification in X-Ray Coronary Angiography

Motivated by the latest results in data-driven image-based analysis, a deep CNN was adopted to solve the coronary angiography image recognition task. The topology of the proposed privacy-preserving CNN is described in Table 5.

To solve the classification problem, the network has to perceive the overall shape of the coronary artery and not just individual pixels. Therefore, to capture features of increasingly higher order, we opted for a deeper convolutional network that expands the model's receptive field and automatically learns relevant features. As the receptive field becomes larger, the layers can capture features with vast semantic meaning. Moreover, to model the global relationship between the extracted features, fully connected layers have been added. Hence, 4 convolutional layers were used to capture relevant features and 2 fully connected layers were used to capture feature dependencies. We gradually increased the number of features by a factor of 2 with the intention of capturing more complex semantic information. To limit overfitting, a dropout layer was employed for regularization, to randomly ignore 25% of the neurons during the training phase. A sigmoid activation function was used in the latest layer to squash the values between [0,1] and formulate the prediction of the model as a probability.

### 6.4. Setup of the Deep Neural Network Models

To train the deep neural network models on ciphertext data, we used the approach described in Algorithm 7. The hyperparameters considered for each experiment are presented in Table 6. To avoid slow convergence and mitigate the chances of vanishing or exploding gradients, especially when using sigmoid or tanh as activation functions, we initialized all weights following Xavier's method [64] with random values chosen from a truncated uniform distribution within $\left[\sqrt{\left(6\right)}/\sqrt{\left(n_{i}+n_{i+1}\right)}\right]$ where *n*_*i*_ and *n*_*i*+1_ represent the number of input and output units of the layer. We chose a variant of gradient descent to update the model's parameter across minibatches of training examples to avoid storing the entire dataset into memory and to speed up training in the context of large datasets.

During the data-driven model optimization, it is necessary to closely monitor the training phase to ensure an optimal performance. An inadequate optimization may lead to a network that can neither model the training data nor generalize on new data. Overfitting and underfitting are the two well-known learning-based problems that greatly affect the performance of the model on an unseen dataset. Therefore, these issues can be avoided by knowing when to stop the training. A commonly adopted strategy to prevent the degradation of the model's performance is to define early stopping criteria based on the error on a validation dataset. More specifically, if the error on a held-out dataset does not improve over time or the gap between the training and validation errors widens, the training can be interrupted. In both strategies, the stopping criteria are set upon the error analysis. Although easily adopted during the training phase on plaintext data, these strategies are becoming highly impractical when operating on ciphertext data. Due to the adopted cryptosystem, and the fact that the error metric is in turn a ciphertext, it cannot be used within a conditional statement. To overcome this limitation, the privacy-preserving models are trained for a predefined number of iterations.

As the overall goal of the study is to assess the feasibility of the deep neural network to operate directly on ciphertext data, i.e., demonstrate that the performance does not drop compared to the plaintext setting, a proper stopping criterion can be predefined. Hence, for usability and simplicity, we have chosen an arbitrarily large number of epochs to conduct the experiments and report the performance.

For each of the tasks, we computed both the unencrypted and the counterpart encrypted version. While the first experiment implies regular training and inference operations, for the encrypted version, the neural network exclusively operates on ciphertext data, with all trainable parameters being completely encrypted. To enable a fair comparison, all networks (plaintext and ciphertext) were trained using the same training strategy, hyperparameters, and initialization method, as outlined in Table 6. Moreover, for consistency, the models trained on both ciphertext and plaintext data started from the same set of initial values.

To measure the performance of the neural network models trained on ciphertext data on the held-out testing set, all evaluation metrics are computed on the decrypted results, where decryption is performed as shown in Algorithm 4.

## 7. Results

To evaluate the performance of the proposed privacy-preserving deep neural network models, two criteria have been examined: reliability and applicability in medical scenarios.

Consequently, for each of the use cases, the performances, and hence the results, of the data-driven models were analyzed by applying the models on both encrypted (ciphertext) and unencrypted (plaintext) data. By comparing the outcomes of the two scenarios, we analyzed and measured the privacy-preserving models' ability to retain the performances.

Besides reliability, another factor that plays an important role in determining the viability of the privacy-preserving models to operate in clinical routines is the runtime. Therefore, a detailed analysis of the runtime was performed, and both the inference and the training times were reported.

The analyses showed that data security can be ensured on the basis of homomorphic encryption, and that, at the same time, deep learning-based data analysis can be efficiently performed. Furthermore, experiments have indicated that in both encrypted and unencrypted versions, the data-driven models are similarly optimized, as outlined in the following.

### 7.1. Performance

**Figure 10:**
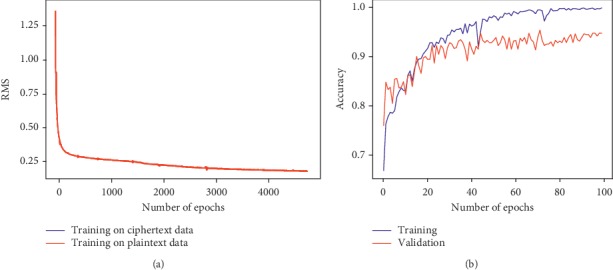
(a) Evolution of the training loss for encrypted and unencrypted networks: differences between the learning curves, caused by floating-point arithmetic, are unnoticeable. (b) Evolution of the accuracy when training on ciphertext data.

To showcase the ability of the network to learn from ciphertext data, the training loss for the regression task, as resulted after decryption, is depicted in Figure 10(a). Similarly, the evolution of the training and validation accuracy of the privacy-preserving CNN model fed with encrypted X-ray coronary angiographies, obtained after decryption, is depicted in Figure 10(b).

The training evolution demonstrates the capability of the proposed method to preserve the correctness of the computations. Moreover, after decryption, the parameters learned by the model when trained on ciphertext data were found to be identical up to machine precision to those learned by the unencrypted model. Therefore, the overall performance of the deep learning models on the held-out testing samples has been proven to be consistent regardless of whether the model was previously trained with or without encryption. Hence, all performance metrics reported hereafter are based on the results obtained by the encrypted deep neural network models.

#### 7.1.1. MNIST Binary Classification

The default metric used to assess the performance of a classifier on the MNIST dataset is given by the absolute accuracy of the classification models, i.e., the percentage of correctly labeled digit images. The unencrypted network achieved a classification accuracy of 98.2% on the testing dataset, which was preserved by the encrypted network. In the MNIST database, the class distribution in the held-out testing set is well balanced across the 10 labels, and hence accuracy can be seen as a reliable metric for assessing the classification performance.

**Figure 11:**
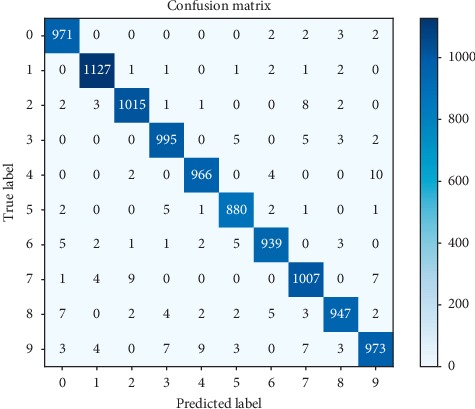
Confusion matrix of the MNIST digit classification task on the test set. The number on the diagonal indicates the number of correctly classified images, while the rest represent the misclassified ones.

For a more detailed evaluation of the performance, we used three additional criteria defined as follows:

- Precision : (TP/(TP+FP))
- Recall : (TP/(TP+FN))
- *F*1 − score : (2 · precision · recall/(precision+recall))

where TP represents the number of images correctly predicted as being positive, TN is the number of images correctly predicted as being negative, FP shows the number of images incorrectly predicted as positive, and FN is the number of images incorrectly predicted as negative. Being a multiclass classification problem, the evaluation metrics were computed following the one-vs-rest strategy. More specifically, to compute the metrics, each label was individually considered positive while the others were set as being negative. The precision, recall, and *F*1-score for each digit class are reported in Table 7. To evaluate the digit recognition performance of the proposed CNN model, we show the confusion matrix in Figure 11.

Although 98.2% is an acceptable accuracy for the MNIST digit recognition task, the proposed model did not reach the reported state-of-the-art accuracy of 99.77%. This was to be expected as the classifier proposed to solve the problem of digit recognition was primarily adopted to assess the correctness of the privacy-preserving computations within a traditional convolutional neural network.

The performance of any neural network model can be generally improved by tuning the architecture of the network or by adopting more favorable activation functions and optimization algorithms. Additionally, training data augmentation techniques, e.g. elastic distortions, can be employed to further decrease the classification error rate, as shown in [80]. However, optimizing the model or the hyperparameters to achieve maximum performance on the MNIST digit recognition task was beyond the scope of this work.

#### 7.1.2. Hemodynamic Analysis

**Figure 12:**
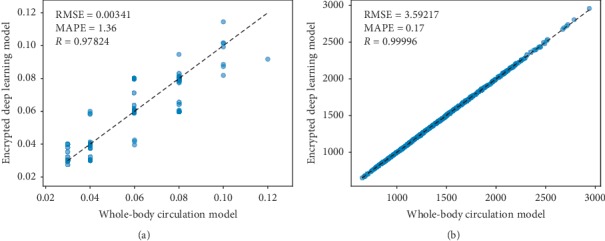
Predicted vs. ground truth. (a) Ratio of proximal to distal resistance in the systemic circulation and (b) systemic resistance.

The performance of the deep neural network model predicting the outputs of the WBC model was evaluated based on the Pearson correlation and the average absolute relative error: Table 8 shows the results.  Figure 12 depicts scatter plots of the predicted vs. measured quantities, having the smallest (ratio of proximal to distal resistance in the systemic circulation) and the largest (systemic resistance) Pearson correlation.

#### 7.1.3. X-Ray Coronary Angiography Classification

For the binary classification problem, i.e., coronary angiography view recognition, the predicted value is the probability of a given input image to represent a right coronary artery (RCA). Thus, the threshold can be chosen to favor a certain problem specific behavior. It introduces flexibility in interpretation, allowing for a trade-off between false negative and false positive findings, by varying a probability threshold. The ROC (receiver operator characteristic) curve is widely used as a way of interpreting the performance of a classifier by describing the trade-off between true and false positives as the threshold is varied.

**Figure 13:**
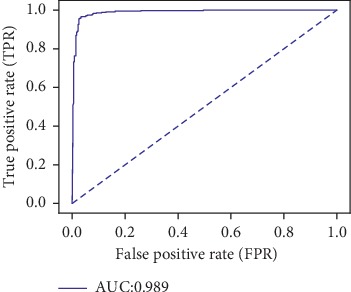
ROC curve of the view classification task in X-ray coronary angiography.

**Figure 14:**
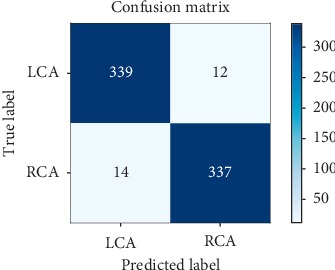
Confusion matrix of the X-ray coronary angiography view classifier.

To assess the accuracy of the coronary angiography view recognition model, the obtained ROC curve is shown in Figure 13.  Table 9 lists the precision, recall, and *F*1-score for both the LCA and RCA labels.  Figure 14 shows the confusion matrix, portraying measures of association between the true labels and the deep neural network predictions of LCA and RCA.

In the angiographic view classification use case, the CNN network trained on ciphertext data classified 96.2% of the samples correctly when evaluated on the held-out testing angiographies. When compared to the unencrypted model, accuracy was identical.

### 7.2. Execution Time

All runtimes reported in the current section were measured on a machine equipped with an Intel(R) Xeon(R) CPU running at 2.10 GHz. The deep learning library which integrates the MORE encryption scheme was written in C++. The library is still under active development, with minimal multithreading support.

A detailed comparison of the runtime for each of the applications is given in Tables 10 and 11. All reported results were obtained by employing data parallelism (8 threads), both during training and inference. Although deep learning models run directly on MORE, homomorphically encrypted data are significantly slower (up to one order of magnitude) during both training and inference, and the scheme is currently outstandingly faster compared to classic fully homomorphic encryption schemes where the difference is of around 6 to 7 orders of magnitude, even when performing very basic algebraic operations.

### 7.3. Security Concerns

Despite the fact that the considered MORE encryption scheme has some advantages in terms of simplicity, clearness, and practicability, with properties tailored to privacy-preserving machine learning, it offers limited security compared to other HE schemes.

The most significant security concern is given by the linear nature of MORE [50, 51], whereas typical encryption schemes are based on strongly nonlinear functions and modular arithmetics over large numbers. This linearity may allow one to determine the secret key by having access to a large enough number of pairs of encrypted and unencrypted values. Otherwise stated, if a sufficiently large number of plaintext-ciphertext data pairs {*C*_*i*_, *m*_*i*_}_*i*=1_^*N*^ are available, the secret key *S* can be determined through an optimization problem. The key search attack can be formulated as finding the best fit of a matrix *S*′ such that (*S*^′^−1^^*C*_*i*_*S*′)_1,1_=*m*_*i*_ for each known pair *s*.

Although less secure than other homomorphic encryption schemes, the MORE scheme remains a viable solution for certain privacy-preserving applications. Consequently, it can be employed in scenarios where the key is never disclosed, e.g., ciphertext data are uploaded to an external computing service while the raw data remain private on the side of the data provider. For example, personal medical data can be uploaded to a dedicated service, i.e. patient-level encryption, that provides a personalized risk factor or other health-related indicators [81].

## 8. Discussion and Conclusions

The growing concern over recent years to preserve the privacy of sensitive patient health information, while promoting the development of personalized medicine, has increased the demand for cryptographic techniques suitable for addressing privacy-related issues in data-driven models.

The current work was focused on designing fully automatic data-driven personalization-based medicine solutions by protecting the integrity of patient health data. A variant of a noise-free matrix-based homomorphic encryption scheme (MORE) was proposed for privacy-preserving computations within deep learning models. Although a homomorphic cryptosystem is governed by a private key to encrypt and decrypt data, it greatly varies from other forms of encryption as it preserves the algebraic properties to allow a variety of operations to be performed directly on the encrypted data (ciphertext data) without requiring access to the decrypted information (plaintext data) or the encryption key.

We have showcased the applicability of incorporating the MORE encryption scheme into deep learning models by tackling three different problems: digit recognition, whole-body hemodynamic analysis, and coronary angiography view classification. The first application focused on a standard benchmarking application from the computer vision realm (MNIST digit recognition) to evaluate the feasibility of a network to operate directly on encrypted data, whereas the latter two models target clinically realistic problems. Therefore, one model was designed to estimate the outputs of a whole-body circulation (WBC) hemodynamic model. The second clinically realistic application was responsible for classifying encrypted X-ray coronary angiography medical images.

For each application, we have addressed both the training and the inference phase and showed that both can be performed on MORE homomorphically encrypted data. The reported results indicate that the proposed solution has great potential: (i) computational results are indistinguishable from those obtained with the unencrypted variants of the deep learning-based applications and (ii) runtimes increase only marginally. The encryption scheme incurs a reasonably small computational overhead and, importantly, allows for operations to be performed directly on floating-point numbers, which represents a critical property for artificial neural networks.

Although the MORE encryption scheme offers lower security compared to standard schemes, it is one of the few schemes with the potential to be used in practical applications. Consequently, it can be incorporated in two deep learning-based scenarios. The first scenario refers to the training of a model with encrypted input-target data and is suited for problems where the requirements are targeted on keeping both the data and the model private. This scenario implies that the unseen data on which the trained model will be applied will have to be encrypted with the same key as the data used for training. The second scenario covers the training of a model on plaintext input-target data, which can then be applied on encrypted data. This facilitates the use of existing deep learning models but also the idea of transferring the knowledge from one problem to another. Although the neural network will not be private, it will be applied on private data, resulting in private results, and more importantly will not be dependent on the key, e.g., if two data owners, with different keys, want to use a deep learning model trained on X-ray coronary angiographies, they can encrypt the data with the private key, feed the encrypted image to the network, and obtain encrypted results which will be interpreted only by the owner of the key.

In conclusion, we showed that a class of homomorphic methods based on linear transformations has a great potential towards facilitating data sharing and outsourcing to third parties for data analytics in regulated areas, but it comes at a cost of weaker security. The security compromise is caused by changing the original homomorphic encryption scheme to enable computations to be performed directly on rational numbers, a fundamental requirement for machine learning models. While the preliminary proposed solution is promising, for practical applications, further improvements are needed to strengthen the security of the scheme.

## Figures and Tables

**Algorithm 1:**
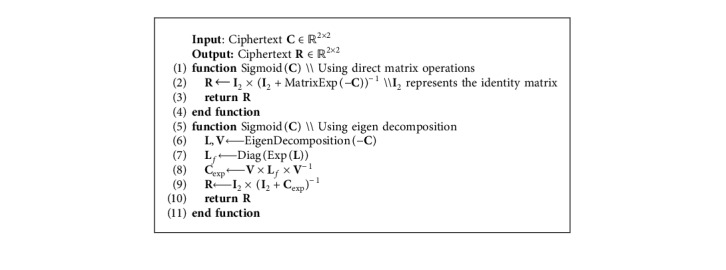
Implementation of the sigmoid function under the MORE encryption scheme.

**Algorithm 2:**
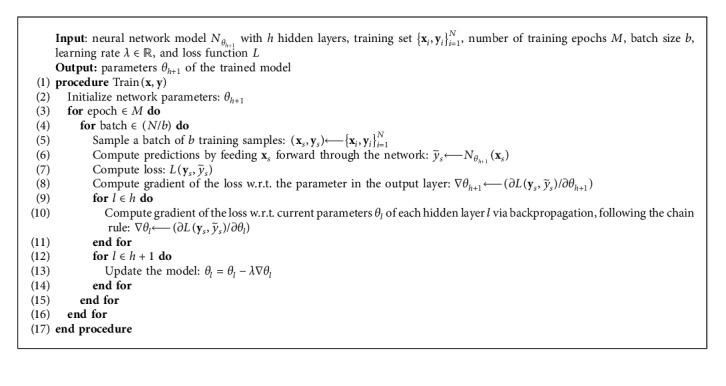
General training algorithm.

**Algorithm 3:**
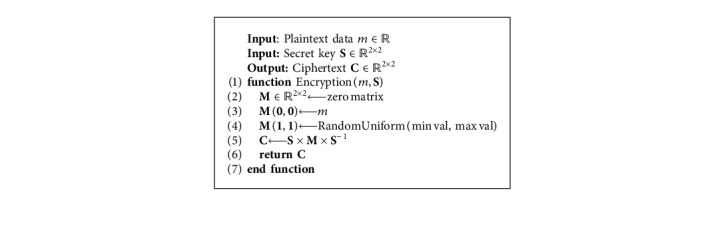
MORE encryption.

**Algorithm 4:**
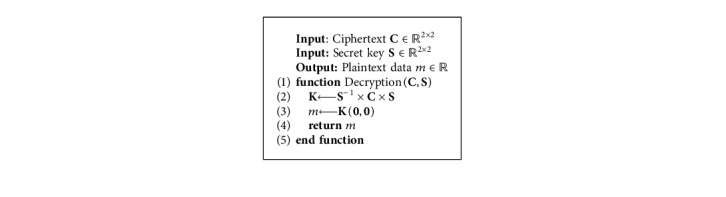
MORE decryption.

**Algorithm 5:**
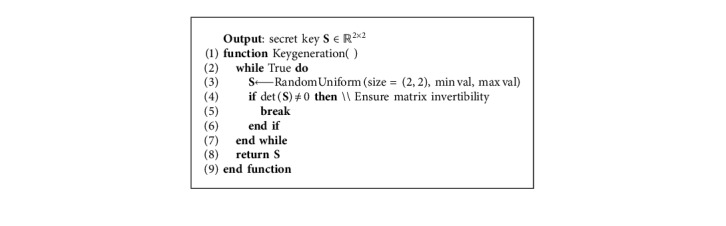
MORE secret key generation.

**Algorithm 6:**
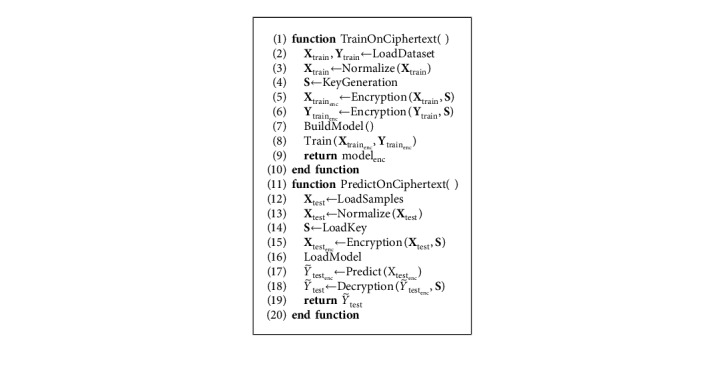
Deep learning-based analysis on ciphertext data.

**Algorithm 7:**
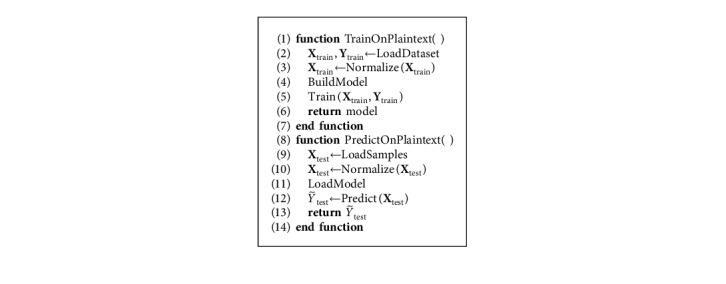
Deep learning-based analysis on plaintext data.

**Table 1:** MORE encryption scheme setup over rational numbers.

Message	Scalar value *m* ∈ *ℝ*
Secret key generation	Invertible matrix *S* ∈ *ℝ*^2×2^
Matrix construction	$M=\left(\begin{array}{cc} m & 0\\ 0 & r \end{array}\right)$, where *r* ∈ *ℝ* is a random parameter
Encryption operation	Encryption(*m*)=*C*=*SMS*^−1^
Decryption operation	Decryption(*C*)=*K*=(*S*^−1^*CS*)
Message recovery	*m*=*K*_(1,1)_

**Table 2:** Overview of databases used for experimental evaluation.

	MNIST	WBC	Angio
Normalization method	Min-max	Mean-std	Min-max
Input dimension	(28, 28)	(1, 9)	(256, 256)
Output dimension	(1, 10)	(1, 12)	(1, 1)
Data augmentation	—	—	Rotation/shifting/zooming
Number of training samples	50000	7000	1996 (7984^*∗*^)
Number of validation samples	10000	1000	680
Number of testing samples	10000	2000	702

^*∗*^After augmentation.

**Table 3:** CNN-MNIST: the topology of the CNN for handwritten digit classification.

Layers	Parameters	Dimensions
Input	—	**(1,28,28)**
Convolution	(8,3,3)	(8,28,28)
Activation (sigmoid)	—	—
Average pooling	(2,2)	(8,14,14)
Convolution	(16,3,3)	(16,14,14)
Activation (sigmoid)	—	—
Average pooling	(2,2)	(16,7,7)
Flatten	—	(784)
Fully connected	100	(100)
Activation (sigmoid)	—	—
Fully connected	10	(10)
Activation (softmax)	—	—

**Table 4:** FCNN-WBC: the topology of the FCNN for hemodynamic analysis.

Layers	Parameters	Dimensions
Input	—	**(9)**
Fully connected	40	(40)
Activation (tanh)	—	—
Fully connected	40	(40)
Activation (tanh)	—	—
Fully connected	40	(40)
Activation (sigmoid)	—	—
Fully connected	12	(12)
Activation (linear)	—	—

**Table 5:** CNN-Angio: the topology of the CNN for view classification in X-ray coronary angiographies.

Layers	Parameters	Dimensions
Input	—	**(1,256,256)**
Convolution	(4,3,3)	(4,256,256)
Activation (sigmoid)	—	—
Average pooling	(2,2)	(4,128,128)
Convolution	(8,3,3)	(8,128,128)
Activation (tanh)	—	—
Average pooling	(2,2)	(8,64,64)
Convolution	(16,3,3)	(16,64,64)
Activation (tanh)	—	—
Average pooling	(2,2)	(16,32,32)
Convolution	(32,3,3)	(32,32,32)
Activation (tanh)	—	—
Average pooling	(2,2)	(32,16,16)
Flatten	—	(8192)
Fully connected	64	(64)
Activation (tanh)	—	—
Dropout	25%	—
Fully connected	—	(1)
Activation (sigmoid)	—	—

**Table 6:** Hyperparameters considered for the learning algorithms.

Hyperparameters	CNN-MNIST	CNN-Angio	FCNN-WBC
Objective function	CE	BCE	MSE
Weight initialization method	Xavier	Xavier	Xavier
Optimizer		Minibatch gradient descent	
Momentum	0.9	0.9	0.9
Learning rate	0.01	0.01	0.01
Batch size	32	16	32
Dropout rate	—	—	25%
Epochs	100	100	4500

**Table 7:** Precision, recall, and *F*1-score of the deep neural network based MNIST digit classification.

Digit	Precision (%)	Recall (%)	*F*1-score (%)
0	97.9	99.0	98.5
1	98.8	99.2	99.0
2	98.5	98.3	98.4
3	98.1	98.5	98.3
4	98.4	98.3	98.4
5	98.2	98.6	98.4
6	98.4	98.0	98.2
7	97.3	97.9	97.6
8	98.3	97.2	97.7
9	97.5	96.4	97.0
*Average*	*98.1*	*98.1*	*98.1*

**Table 8:** Results of the deep neural network for real-time hemodynamic analysis on the testing dataset.

Circulation	Parameters	MAPE (%)	Pearson correlation (%)
Systemic	Dead volume	7.03	0.9997
	Time at max. elastance	0.13	0.9995
	Resistance	0.17	0.9999
	Compliance	2.45	0.9867

Pulmonary	Dead volume	9.88	0.9991
	Time at max. elastance	0.10	0.9994
	Resistance	0.32	0.9998
	Compliance	0.67	0.9983

**Table 9:** Precision, recall, and *F*1-score of deep neural network for hemodynamic analysis.

Label	Precision (%)	Recall (%)	*F*1-score (%)
LCA	96.0	96.5	96.3
RCA	96.5	96.0	96.2
*Average*	*96.2*	*96.2*	*96.2*

**Table 10:** Runtime analysis: mean values and standard deviation of the encrypted and plaintext CNNs for MNIST digit recognition.

Operation	Runtime (s) on ciphertext data	Runtime (s) on plaintext data	Encrypted-unencrypted ratio
Data encryption and key generation	2.44±0.016	—	—
Training (1 epoch)	444.59±8.53	12.98±1.17	34.25
Data encryption	0.39±0.009	—	—
Inference (10 K images)	20.42±0.32	0.54±0.08	37.81
Data decryption	0.001±0.0005	—	—

**Table 11:** Runtime analysis: mean values and standard deviation of the encrypted and plaintext networks for the two personalized medicine use cases.

Task	Operation	Runtime (s) on ciphertext data	Runtime (s) on plaintext data	Encrypted-unencrypted ratio
Angiographic view classification	Training (1 epoch)	1075.47 ± 45.54	34.48 ± 1.12	31.19
	Inference (702 images)	26.36 ± 1.98	0.8 ± 0.06	32.95
Whole-body circulation hemodynamic analysis	Training (1 epoch)	0.66 ± 0.09	0.021 ± 0.001	31.4
	Inference (2000 samples)	0.102 ± 0.01	0.006 ± 0.0009	17

## Data Availability

The MNIST database used to validate the methodology is available at http://yann.lecun.com/exdb/mnist/. The coronary angiography data used to support the findings of this study have not been made available because they were acquired in a research grant (heart.unitbv.ro) which does not allow the publication of the data.
